# Beef Tea and Meat Preparations

**Published:** 1902-02-08

**Authors:** Marcus R. P. Dorman


### THE INSTITUTIONAL WORKSHOP.

### BEEF TEA AND MEAT PREPARATIONS.

By MARCUS R. P. DORMAN, M.D.

### III.—NOURISHING MEAT PREPARATIONS.

MANY preparations now on the market undoubtedly possess great nourishing properties, and in this respect differ both from beef tea and pure extracts. They are manufactured in various ways, which roughly can be described under three headings.

*a.* Preparations in which the meat is peptonised before the extract is made. By this means the peptones which are formed are taken into solution as well as the flesh bases. Armour and Co. claim that their soluble beef "contains all the valuable properties of beef in a partly pre-digested form." Brand and Co.'s peptones can also be included in this class.

*b.* Preparations in which beef fibre or finely-ground "beef meal" is added to the extract proper. Such material is insoluble, and requires to be digested in the stomach before it becomes available for the repair of tissue waste. It does not form part of the real extract, and has the same nourishing value as an equal weight of ordinary lean beef, but has the advantage that it exists as a fine powder, and thus is more easily acted upon by the gastric juices. Armour and Co.'s Vigoral, Bovril, Invalid Bovril,[1] and Brand and Co.'s Fibrous Beef Tea come under this heading.

*c.* Preparations in which the nourishing substances are directly recovered from the meat. The meat juices and Mamesin as prepared by the Myosin Albumin Company can be quoted as examples.

The main difference between these preparations and beef tea or extracts is that they contain large quantities of the albumins, globulins, and paraglobulins of muscle plasma, which are nutritive, formative, and reparative, as well as the flesh bases, which are stimulants. Nourishing proteids exist largely in meat and form the chief part of the residue which is often thrown away after the "goodness" is supposed to have been extracted in the process of making beef tea. They are allied to the proteid tissues of the body and possess complex functions as heat-producing and motor agents, so that when muscle or nerve tissue is actively used it is necessary to ingest a sufficient quantity daily to replace the waste products which are passing through the creatinoid stages to become urea. Milk also contains albumin in the form of casein, which is an excellent food, but many adults find it difficult to digest. These meat preparations are therefore particularly suitable for all cases which require not only a stimulating extract, but also nourishment in a liquid and easily digestible form, and should therefore be preferred to beef tea in all long-standing illnesses, slow convalescences, wasting diseases, and other conditions in which a building up of the tissues is of greater importance than a sudden manifestation of energy. It is not our intention to attempt to describe or compare the merits of these various preparations, but in order that some opinion may be formed as to the amount to be used in making solutions for patients we have constructed the following table showing the percentage of water in each, according to the authority quoted.

| Name of Preparation. | Water. per cent. | Analytical Authority. |
|---|---|---|
| Armour's Extract ... | ... 21·9 ... | Inland Revenue Laboratory, Ottawa. |
| Armour's Beef Juice | ... 74·10 ... | Professor John Attfield, F.R.S. |
| Bovril... ... ... | ... 37·2 ... | Inland Revenue Laboratory, Ottawa. |
| Invalid Bovril ... | ... 21·16 ... | Tankard (Allen's Commercial Organic Analysis). |
| Brand's Beef Tea tabules | ... 15·38 ... | *Lancet*, Jan. 22nd, 1898. |
| Brand's Meat Juice... | ... 60·00 ... | *Lancet*, Jan. 7th, 1893. |
| Lemco ... ... | ... 17·72 ... | *Science Siftings*, Feb. 3rd, 1900. |
| Oxo ... ... ... | ... 36·64 ... | *Science Siftings*, Oct. 19th, 1901. |
| Ma Me Co ... ... | ... 86·04 ... | *Lancet*, Sept. 28th, 1901. |
| Mamesin ... ... | ... 81·24 ... | Ibid. |
| Valentine's ... ... | ... 54·6 ... | Inland Revenue Laboratory, Ottawa. |
| Vigoral ... ... | ... 43·6 ... | Ibid. |

A glance at this table shows that great care must be used in ordering, for it is obvious that a teaspoonful of some of these preparations would contain a considerable amount more water and less solids than the same amount of others and if we wish to use them scientifically it is very necessary to know how much extract and nourishment we are giving Although this can only be estimated exactly by referring to the full analyses, the amount of water is probably a sufficient rough guide for clinical purposes. Apart from the scientific superiority of such preparations over domestic beef tea, the flavour and appearance are such as to be tempting to the most jaded appetite. Whoever has experienced the misfortune of a lengthy illness knows how terribly the same monotonous solution of beef palls, until finally it is merely swallowed because it is regarded as an unpleasant but necessary drug. Bovril, on the other hand, is decidedly pleasant. Oxo has the appearance and flavour of a first-class soup, and with such a number of preparations to choose from the patient can have a varied diet every day. Mamesin contains the high proportion of 11·73 per cent. of albumen, and can be added to milk puddings since it can be made absolutely tasteless.

The nourishing preparations are, then, particularly suitable for cases of prolonged debility after illness, and for feeding up in cases of phthisis, neurasthenia, heart disease, enteric, or gastric troubles where it is difficult to digest meat. The combination of the extractives and nourishing properties of meat enables the feeblest system not only to show an improved vitality, but also to build up its wasted tissues. Chemically, physiologically, and clinically they are superior in every respect to beef tea. The old idea that there is any nourishment in the latter is already dead; it is becoming every day more widely known that the simple extracts are merely stimulants, and every day there appear new nourishing preparations whose number is legion and whose utility is as great as their number. It now remains simply to prove that their cost is considerably less than the domestic solution of beef.

[1] The chief difference between Bovril and Invalid Bovril is that the latter contains more nourishing substances.

